# Enroller Experience and Parental Familiarity of Disease Influence Participation in a Pediatric Trial

**DOI:** 10.5811/westjem.2021.4.54647

**Published:** 2021-09-02

**Authors:** Jeff E. Schunk, Kammy K. Jacobsen, Dilon Stephens, Amy Watson, Cody S. Olsen, T. Charles Casper, Nicole S. Glaser, Nathan Kuppermann

**Affiliations:** * University of Utah School of Medicine, Department of Pediatrics, Salt Lake City, Utah; † University of California Davis Health, Department of Pediatrics, Sacramento, California; ‡ University of California Davis Health, Department of Emergency Medicine, Sacramento, California

## Abstract

**Introduction:**

Acquiring parental consent is critical to pediatric clinical research, especially in interventional trials. In this study we investigated demographic, clinical, and environmental factors associated with likelihood of parental permission for enrollment in a study of therapies for diabetic ketoacidosis (DKA) in children.

**Methods:**

We analyzed data from patients and parents who were approached for enrollment in the Pediatric Emergency Care Applied Research Network (PECARN) Fluid Therapies Under Investigation in DKA (FLUID) trial at one major participating center. We determined the influence of various factors on patient enrollment, including gender, age, distance from home to hospital, insurance status, known vs new onset of diabetes, glycemic control (hemoglobin A1c), DKA severity, gender of the enroller, experience of the enroller, and time of enrollment. Patients whose parents consented to participate were compared to those who declined participation using bivariable and multivariable analyses controlling for the enroller.

**Results:**

A total of 250 patient/parent dyads were approached; 177 (71%) agreed to participate, and 73 (29%) declined. Parents of patients with previous episodes of DKA agreed to enroll more frequently than those with a first DKA episode (94.3% for patients with 1–2 previous DKA episodes, 92.3% for > 2 previous episodes, vs 64.9% for new onset diabetes and 63.2% previously diagnosed but no previous DKA). Participation was also more likely with more experienced enrollers (odds ratio [95% confidence interval] of participation for an enroller with more than two years’ experience vs less than two years: 2.46 [1.53, 3.97]). After adjusting for demographic and clinical factors, significant associations between participation and both DKA history and enroller experience remained. Patient age, gender, distance of home from hospital, glycemic control, insurance status, and measures of DKA severity were not associated with likelihood of participation.

**Conclusion:**

Familiarity with the disease process (previously diagnosed diabetes and previous experience with DKA) and experience of the enroller favorably influenced the likelihood of parental permission for enrollment in a study of DKA in children.

## INTRODUCTION

Research involving human subjects requires informed consent to be obtained from participants. In human subjects research involving children, consent is generally obtained from parents or guardians. The decision-making process involving consent is complicated, and factors associated with participation in pediatric clinical research are poorly understood. These factors may include attributes intrinsic or extrinsic to the participants, including environmental factors. Understanding factors associated with successful enrollment has important implications for research. Reluctance of parents to involve their children in research studies may limit the researcher’s ability to enroll sufficient numbers of patients for optimal study validity. Difficulties in recruiting an adequate number of participants may impact study feasibility, extend study duration, and increase costs. Study generalizability may also be impacted by selection bias if patients with specific characteristics are more likely to enroll. Understanding factors influencing the consent process is important for maximizing participation in future trials.

Previous studies have explored the consent process using surveys, interviews, hypothetical scenarios, focus groups, and comparisons between enrolled vs non-enrolled patients.^1^–^10^ A Cochrane review concluded that “it is not possible to predict the effect most interventions will have on recruitment.”^11^ Most studies have focused on parental characteristics (eg, education level or socioeconomic status) or their attitudes and beliefs about research in general (eg, trust in the investigators or attitudes about research involving children). We hypothesized that factors beyond parental characteristics and attitudes, such as aspects of the enrollment experience, the enroller, and characteristics of the patient’s illness, might play a role in the decision-making process. We investigated demographic, clinical, and environmental factors associated with parental permission to enroll in the Pediatric Emergency Care Applied Research Network’s (PECARN) Fluid Therapies Under Investigation in DKA (FLUID) trial.^12^,^13^

## METHODS

### Overview of Clinical Trial

The current substudy of the PECARN FLUID study was performed at a single institution, Primary Children’s Hospital (PCH), between 2011–2015. The hospital was one of 13 participating sites in a large, multicenter pediatric clinical trial, the PECARN FLUID study.^13^ The FLUID study compared intravenous (IV) fluid regimens for treatment of DKA and demonstrated that there were no differences in neurological outcomes for children rehydrated with more rapid vs slower fluid infusion rates, nor for 0.9% NaCl vs 0.45% NaCl rehydration solutions. The details of the study design and objectives are outlined elsewhere.^12^,^13^ Patients were eligible for the FLUID study if they were younger than 18 years, presented with DKA requiring IV insulin, and had a Glasgow Coma Scale (GCS) score of 12 or higher. The study included children with previously diagnosed and newly diagnosed type 1 diabetes (T1D). Only families who were English- or Spanish-speaking were considered for enrollment. Hospital-based interpreting services were used for Spanish-speaking families, as needed.

### Overview of Enrollment Process

The study was briefly introduced to potential participants by a pediatric or general emergency physician, or pediatric emergency medicine fellow. Families that were willing to consider participation after this brief introduction were then approached by one of 12 trained research assistants (“enrollers”). Enroller training included standardized training in bioethics and good clinical practice as well as study-specific training and mock practice sessions prior to approaching any participants. All enrollers were paid employees of the university. Those with less than two years experience were classified as research assistants and generally worked 20 hours per week or less. Those with more than two years experience were classified as research coordinators and were more likely to work full time (more than 30 hours per week). A total of 12 enrollers (10 female, 2 male) were used during the course of the study with three of them moving from research assistant to research coordinator during the study period. Parents or guardians reviewed the consent form, and questions were answered by the enroller. Questions unable to be answered by the enroller were answered by the attending physician or pediatric emergency medicine fellow.

### Data Abstraction

The current study was an unplanned secondary analysis that involved data abstracted from the health records, the enrollment debriefing form (used by enrollers to document interactions with the parents of potential study patients), and the FLUID trial dataset. Enrollment debriefing forms included information about the consent experience such as questions asked by parents/participants, the amount of time spent conducting consent, the outcome of the consent process, and the enroller’s comments regarding the consent process. Patient demographic factors including gender, age, distance of patient’s home from hospital, and insurance status (categorized as insured, uninsured, government insurance) were recorded. Distance of patient’s home from the hospital was calculated using an average distance based on ZIP code and Google Maps estimate. Calculations of distance traveled to the hospital assumed that all patients would be traveling by car, given the very limited public transportation in the region of the study site. Other clinical factors (new onset T1D or previously diagnosed T1D, hemoglobin A1c (HbA1c) in known T1D patients, biochemical indicators of DKA severity, GCS score at presentation), and environmental factors (gender of enroller, experience of enroller, time of day) were recorded.

The sample size calculations for the parent study are described in detail in previous publications.^12^,^13^ Individual sites did not have specific quotas to contribute but rather the study continued until the necessary sample size was achieved across all sites. We included all available data from PCH in this analysis.

### Statistical Analyses

We estimated the overall consent rate and consent rates for subsets of patients based on patient and enroller characteristics. We estimated means and standard deviations of continuous characteristics for patients whose parents consented to participate and those who declined. We compared patient and enroller characteristics between groups using logistic regression models fit using the generalized estimating equation (GEE) method.^14^ Multivariable associations between parent/patient participation and both patient and enroller characteristics were additionally estimated using multivariable logistic GEE models. We chose factors for inclusion in multivariable models based on bivariable associations (*P* <0.20). All GEE models assumed exchangeable working correlation among subjects approached by the same enroller. We estimated unadjusted and adjusted odds ratios (OR) and 95% confidence intervals (CI) and used a significance level of 0.05 for all statistical tests. We performed analyses using SAS/STAT software version 9. (SAS Institute, Inc., Cary, NC).

The study was approved through the University of Utah School of Medicine and PCH institutional review boards as a sub-study to the original, parent FLUID study. Participants were not re-approached for additional consent to collect data for this sub-analysis.

## RESULTS

Among 250 patients approached for participation in the FLUID study at PCH, guardians consented to participate in 177 cases (71%, Table 1, Figure). Patient age, gender, insurance status, distance of the patient’s home from the hospital, severity of DKA and mean HbA1c (for previously diagnosed T1D patients) were not significantly associated with enrollment decisions in bivariable analyses (Table 2). Patients with one or more previous episodes of DKA were more likely to participate than new onset or previously diagnosed T1D patients with first episodes of DKA (OR [95% CI] comparing 1–2 previous DKA episodes to new onset patients: 8.3 [3.0, 22.8]; and comparing three or more previous DKA to new onset patients: 5.8 [1.6, 21.1]. Greater experience of the enroller (more than two years’ experience enrolling patients in clinical studies) also favorably influenced participation rates: 2.5 [1.5, 4.0]. The gender of the enroller and time of day did not significantly influence participation. In a multivariable model (adjusting for enroller experience, diabetes and DKA history, and initial pH), diabetes and DKA history and enroller experience remained significantly associated with enrollment (Table 2). In that analysis, greater enroller experience was associated with 2.4 times the odds of participation (95% CI, 1.4, 4.3). Previous episodes of DKA also remained significantly associated with increased adjusted odds of enrollment.

## DISCUSSION

Our data suggest that familiarity with the disease process substantially influences decisions about enrollment in clinical research. We found that children with previously diagnosed T1D and previous episodes of DKA were enrolled at significantly higher rates than those with first DKA episodes and those with newly diagnosed T1D. Study participation was also more likely with more experienced enrollers. Gender of the enroller, time of day, patient age, gender distance from home to hospital, glycemic control, insurance status, and measures of DKA severity did not significantly influence likelihood of enrollment. To our knowledge, this study is the first to document that familiarity with the disease process is a strong predictor of likelihood of enrollment in a research study. Furthermore, some factors that might intuitively seem likely to influence research participation, such as the severity of the child’s illness or the differences in the burden to the family resulting from travel to the hospital, did not have a substantial effect.

Parental decisions to allow child participation in prospective clinical trials is complicated by perceptions regarding “experimentation,” attitudes toward research, altruism, desires for the best care for their child, and other personal beliefs and attributes. The research setting often plays an important role in decision-making. Parents are more likely to endorse research in the non-emergency setting and perceive emergency research as more risky.^8^ Parents of children with oncologic and other life-threatening conditions tend to view the risks of research involving these conditions less negatively than research involving healthy children.^15^ Parents’ characteristics also may influence decision-making. Higher levels of benevolence and altruism, higher levels of trust, introversion, lower self-esteem, and less decisional anxiety and uncertainty are associated with higher likelihood of research participation.^2^,^3^,^5^,^16^ Better understanding of randomization and of the medical system in general are also associated with higher rates of participation.^2^,^3^

Environmental and study-related factors may also influence study enrollment. Clarity of information provided, adequacy of time to make the decision, and the amount of privacy provided have been previously found to influence enrollment decisions.^3^ In the current study, enroller experience significantly impacted likelihood of enrollment. In a survey of parents of children with T1D, trust in the provider, comfort with consent by proxy, and ease of understanding of the information were important factors influencing study enrollment.^7^ These findings are consistent with our results as more experienced enrollers typically have increased familiarity and comfort with study details and methods, contents of informed consent form, and family interactions during a time of medical crisis. These factors would allow for a more relaxed, comfortable, and informative interaction, increasing the likelihood of enrollment. Notably, our study highlighted that parents who have previous experience with the condition being studied (DKA or T1D in this study) are more likely to participate. Factors that might influence parents’ perceptions of their children’s vulnerability (such as severity of illness and younger age) did not appear to impact enrollment. Neither did longer distance from home to hospital (with more inconvenient follow-up) affect likelihood of participation. These findings have important implications for estimating participation rates in future research.

## LIMITATIONS

The study is subject to several limitations. First, the focus of the PECARN FLUID trial was to investigate neurological outcomes of different rehydration strategies in children with DKA. The study did not include assessments of parental beliefs or attitudes about research, and data about some demographic variables that might be of interest, such as parents’ age, gender, and educational level, were not recorded. It is therefore possible that factors not assessed in this study also contributed to parental decision-making regarding participation. The relatively small sample size of the study also limited our ability to assess some variables that might exert a more modest influence on parental decision-making.

In addition, the current analysis involved a single study center with a narrower cultural, socioeconomic, and ethnic range than that of the full cohort of patients in the multicenter study. The patient population in this sub-study was predominantly White and non-Hispanic. It is possible that results may differ in populations with different characteristics, although most patients with T1D meet this racial/ethnic profile.^17^ The number of research personnel approaching patients for enrollment also was relatively small, limiting our ability to detect some associations between enroller characteristics and likelihood of consent. In addition, the data from this study pertains to patients with exacerbations of a chronic condition. Factors associated with likelihood of enrollment in studies pertaining to conditions that are unlikely to recur may differ from those identified in this study. Finally, the current study was federally funded and enrollers were highly trained. Factors influencing enrollment in unfunded studies, which may involve less rigorous enroller training and be viewed with less confidence by parents considering enrollment, might differ.

## CONCLUSION

Our study underscores the importance of parental familiarity with the disease being studied and experience of the enroller in influencing parental decisions regarding research participation of children. These data have important implications for future pediatric clinical trial designs and expectations regarding enrollment to promote successful completion of study objectives. Additional studies involving observation of recruitment/enrollment and open-ended questions about opinions regarding research participation could be helpful to further explore the observed phenomena.

## Figures and Tables

**Figure:**
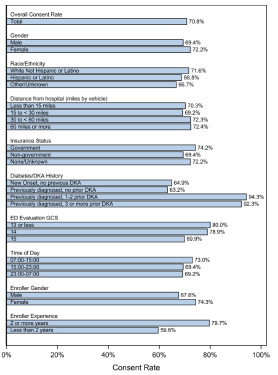
Consent rates by patient and enroller characteristics. *DKA*, diabetic ketoacidosis; *ED*, emergency department, *GCS*, Glasgow Coma Scale.

**Table 1:** Patient demographic and clinical characteristics and enroller characteristics.

	Overall N (%) or Mean (SD)	Declined N (%) or Mean (SD)	Consented N (%) or Mean (SD)
Total	250	73	177
Age at screening: mean (SD)	10.9 (4.42)	10.5 (4.60)	11.1 (4.34)
Gender			
Male	124 (49.6%)	38 (52.1%)	86 (48.6%)
Female	126 (50.4%)	35 (47.9%)	91 (51.4%)
Race/ethnicity			
White, not Hispanic or Latino	194 (77.6%)	55 (75.3%)	139 (78.5%)
Hispanic or Latino	32 (12.8%)	10 (13.7%)	22 (12.4%)
Other/unknown	24 (9.6%)	8 (11.0%)	16 (9.0%)
Distance from hospital (miles)			
Less than 15 miles	91 (36.4%)	27 (37.0%)	64 (36.2%)
15 to <30 miles	65 (26.0%)	20 (27.4%)	45 (25.4%)
30 to <60 miles	65 (26.0%)	18 (24.7%)	47 (26.6%)
60 miles or more	29 (11.6%)	8 (11.0%)	21 (11.9%)
Insurance status			
Government	62 (24.8%)	16 (21.9%)	46 (26.0%)
Non-government	170 (68.0%)	52 (71.2%)	118 (66.7%)
None/unknown	18 (7.2%)	5 (6.8%)	13 (7.3%)
Diabetes/DKA history*			
New onset, no previous DKA	148 (59.9%)	52 (74.3%)	96 (54.2%)
Previously diagnosed, no previous DKA	38 (15.4%)	14 (20.0%)	24 (13.6%)
Previously diagnosed, 1–2 previous DKA	35 (14.2%)	2 (2.9%)	33 (18.6%)
Previously diagnosed, 3 or more previous DKA	26 (10.5%)	2 (2.9%)	24 (13.6%)
12 month average HbA1c (known T1D only): mean (SD)*	10.6 (2.3)	10.6 (2.6)	10.6 (2.3)
Initial glucose (mg/dL): mean (SD)	531 (154.8)	548 (175.6)	524 (145.2)
Initial pH: mean (SD)	7.17 (0.11)	7.18 (0.10)	7.17 (0.11)
Initial BUN (mg/dL): mean (SD)	16.6 (7.4)	16.6 (8.7)	16.6 (6.9)
ED Evaluation GCS score			
13 or less	5 (2.0%)	1 (1.4%)	4 (2.3%)
14	19 (7.6%)	4 (5.5%)	15 (8.5%)
15	226 (90.4%)	68 (93.2%)	158 (89.3%)
Time of day			
07:00–15:00	100 (40.0%)	27 (37.0%)	73 (41.2%)
15:00–23:00	124 (49.6%)	38 (52.1%)	86 (48.6%)
23:00–07:00	26 (10.4%)	8 (11.0%)	18 (10.2%)
Enroller gender*			
Male	74 (30.2%)	24 (35.3%)	50 (28.2%)
Female	171 (69.8%)	44 (64.7%)	127 (71.8%)
Enroller experience**			
≥ 2 years	187 (79.9%)	38 (66.7%)	149 (84.2%)
< 2 years	47 (20.1%)	19 (33.3%)	28 (15.8%)

* Diabetes/DKA history was missing for three declined, HbA1c was missing for six previously diagnosed consented, and enroller gender was missing for five declined parent/patient dyads.

*SD*, standard deviation; *DKA*, diabetic ketoacidosis; *HbA1c*, hemoglobin A1c; *T1D*, type 1 diabetes; *ED*, emergency department; *mg/dL*, milligrams per deciliter; *BUN*, blood urea nitrogen; *ED*, emergency department; *GCS*, Glasgow Coma Scale.

** For 11 patients, the family declined to hear more information about the study after the initial introduction to the study by the physician; enroller experience was undocumented in another five parent/patient dyads who declined consent; these 16 were not included in analyses of enroller characteristics or in multivariable models.

**Table 2:** Unadjusted and adjusted odds-ratio estimates for successful enrollment.*

Characteristic	Odds ratio (95% CI)	Adjusted odds ratio (95% CI)†
Age at screening (per 1 year increase)	1.03 (0.96, 1.11)	
Gender		
Male	[Reference]	
Female	1.19 (0.82, 1.72)	
Race/Ethnicity		
White, not Hispanic or Latino	[Reference]	
Hispanic or Latino	0.87 (0.42, 1.83)	
Other/unknown	0.80 (0.42, 1.53)	
Distance from hospital (miles)		
Less than 15 miles	[Reference]	
15 to <30 miles	0.95 (0.48, 1.86)	
30 to <60 miles	1.08 (0.61, 1.90)	
60 miles or more	1.12 (0.63, 2.02)	
Insurance status		
Government	1.16 (0.78, 1.73)	
Non-government	[Reference]	
None/unknown	1.13 (0.35, 3.63)	
Diabetes/DKA History		
New onset, no previous DKA	[Reference]	[Reference]
Previously diagnosed, no previous DKA	0.91 (0.53, 1.58)	1.05 (0.49, 2.23)
Previously diagnosed, 1–2 previous DKA	8.29 (3.01, 22.82)	11.26 (2.55, 49.60)
Previously diagnosed, 3 or more previous DKA	5.76 (1.57, 21.13)	5.32 (1.37, 20.70)
12-month average HbA1c result (known T1D only) (per 1% increase)	1.01 (0.83, 1.24)	
Initial glucose (per 100 mg/dL increase)	0.92 (0.83, 1.01)	0.99 (0.86, 1.15)
Initial pH (per 0.1 increase)	0.86 (0.72, 1.01)	0.86 (0.69, 1.07)
Initial BUN (per 1 mg/dL increase)	1.00 (0.97, 1.03)	
ED evaluation GCS score		
13 or less	1.82 (0.33, 10.04)	
14	1.48 (0.60, 3.65)	
15	[Reference]	
Time of day		
07:00–15:00	1.21 (0.57, 2.58)	
15:00–23:00	1.09 (0.49, 2.44)	
23:00–07:00	[Reference]	
Enroller gender		
Male	[Reference]	
Female	1.39 (0.71, 2.74)	
Enroller experience		
2 or more years	2.46 (1.53, 3.97)	2.42 (1.37, 4.28)
Less than 2 years	[Reference]	[Reference]

* Odds ratios and 95% CIs were estimated from logistic regression models fit using generalized estimating equations and controlled for correlation between subjects approached by the same enroller using an exchangeable working correlation. Odds ratios >1 signify increased odds of parent/patient consent to participate.

† Multivariable model variables were selected based on unadjusted associations with P <0.20.

*CI*, confidence interval; *DKA*, diabetic ketoacidosis; *HbA1c*, hemoglobin A1c; *T1D*, type 1 diabetes; *ED*, emergency department; *mg/dL*, milligrams per deciliter; *BUN*, blood urea nitrogen; *ED*, emergency department; *GCS*, Glasgow Coma Scale.
